# Patterns Associated with Adult Mandibular Fractures in Southern Taiwan—A Cross-Sectional Retrospective Study

**DOI:** 10.3390/ijerph14070821

**Published:** 2017-07-24

**Authors:** Ko-Chien Lin, Shu-Hui Peng, Pao-Jen Kuo, Yi-Chun Chen, Cheng-Shyuan Rau, Ching-Hua Hsieh

**Affiliations:** 1 Department of Plastic and Reconstructive Surgery, Kaohsiung Chang Gung Memorial Hospital and Chang Gung University College of Medicine, Ta-Pei Road, Niao-Song District, Kaohsiung City 833, Taiwan; kochienlin@gmail.com (K.-C.L.); pshui@cgmh.org.tw (S.-H.P.); bow110470@gmail.com (P.-J.K.); libe320@yahoo.com.tw (Y.-C.C.); 2 Department of Neurosurgery, Kaohsiung Chang Gung Memorial Hospital and Chang Gung University College of Medicine, Ta-Pei Road, Niao-Song District, Kaohsiung City 833, Taiwan; ersh2127@cloud.cgmh.org.tw

**Keywords:** trauma, mandibular fracture, motorcycle accident, helmet

## Abstract

*Purpose:* This study aimed to determine the patterns associated with adult mandibular fractures from a Level-I trauma center in southern Taiwan. *Methods:* The data of adult trauma patients admitted between 1 January 2009 and 31 December 2014 were retrieved from the Trauma Registry System and retrospectively reviewed. Fracture site and cause of injury were categorized into groups for comparison, and corresponding odds ratios (ORs) and 95% confidence intervals (CIs) were obtained by multivariate logistic regression. *Results:* Motorcycle accidents were the most common cause of mandibular fractures (76.3%), followed by falls (10.9%), motor vehicle accidents (4.8%), and being struck by/against objects (4.5%). Of the 503 cases of mandibular fractures, the condylar neck and head were the most common sites (32.0%), followed by the parasymphysis (21.7%), symphysis (19.5%), angle and ramus (17.5%), and body (9.3%). The location of mandibular fractures in patients who had motorcycle accidents was similar to that in all patients. Motor vehicle accidents resulted in a significantly higher number of body fractures (OR 3.3, 95% CI 1.24–8.76, *p* = 0.017) and struck injury in a significantly higher number of angle and ramus fractures (OR 3.9, 95% CI 1.48–10.26, *p* = 0.006) compared to motorcycle accidents. The helmet-wearing status and body weight were not associated with the location of mandibular fractures in motorcycle accidents. *Conclusions:* Our study revealed that the anatomic fracture sites of mandible were specifically related to different etiologies. In southern Taiwan, motorcycle accidents accounted for the major cause of mandibular fractures and were associated with the condylar neck and head as the most frequent fracture sites. In contrast, motor vehicle accidents and struck injuries tended to cause more body fracture as well as angle and ramus fracture compared to motorcycle accidents. Furthermore, the status of helmet-wearing and body weight were not associated with the location of mandible fractures caused by motorcycle accidents.

## 1. Background

Mandibular fracture is one of the most common trauma injuries [1]. In Taiwan, among 6013 patients of a 15-year retrospective study, the mandibles were the second most commonly fracture sites (24.7%), followed by the nasal bones (22.8%), but less than the malar and maxillary bones (48.0%) [2]. A review of the literature indicates that the most common causes of a mandibular fracture are interpersonal conflicts or motor vehicle collisions [3,4,5]. In contrast, in an evaluation of epidemiological data from two European centers, fractures resulted mainly because of assaults and falls [6]. Notably, various fracture sites of the mandible are indicated in different studies [6,7,8]. Christopher et al. reported that the leading anatomic fractures were most commonly located at the angle (27%), followed by the symphysis (21.3%), condyle and subcondyle (18.4%), and body (16.8%) [8]. Fridrich et al. described that the mandibular angle (28.5%) is the most common site of fractures, followed by the mandibular symphysis (21.4%) [4], while King el al. described that parasymphyseal fractures were the most frequent, followed by condyle/head, body, and angle fractures [5]. Obviously, a strong relationship exists between the cause of trauma and anatomic sites of mandibular fractures [4,8,9]. In an analysis of 4143 mandibular fractures, the mandibular angle was the most common fracture site (31.5%) in low-velocity blunt injuries (interpersonal conflict, falls, sports-related injury, and struck by falling object), while high-velocity blunt injuries (motor vehicle collisions, motorcycle collisions, and collisions between a motor vehicle and a pedestrian) resulted in a larger number of condylar fractures (25.4%), followed by symphysis fractures (22.8%) [8]. Zhou et al. reported that falls at a ground level and from a height were associated with a 9.64-fold and 9.17-fold risk of mandibular condylar fractures, respectively, while no significant relationship existed between assault and condylar fractures [9]. Bolagi et al. found that the increasing trend of interpersonal violence caused a shift in the location of mandible fractures from symphyseal–condylar complex to angle–body/parasymphyseal complex [10].

In Taiwan, traveling by motorcycle has been a common part of daily life and remains a crucial mode of transportation [11,12], and as a result, motorcyclists comprise a major portion of the trauma population [13,14,15]. Therefore, the cause and location of mandibular fractures in Taiwan may differ from that observed in Western countries and should be re-evaluated, especially considering that almost all motorcycles are forbidden on highways in Taiwan and most motorcycle traffic accidents occur in relatively crowded streets and at low velocities [13,16,17]. Under the hypothesis that the anatomic fracture sites of mandible are specifically related to different trauma mechanisms, this study examined patients with mandibular fractures in Taiwan with a specific focus on motorcycle accidents as a cause and provides an epidemiological picture from a Level-I trauma center in southern Taiwan.

## 2. Methods

The study was conducted at Kaohsiung Chang Gung Memorial Hospital, a 2400-bed facility and Level-I trauma center that provides care to trauma patients, primarily from the southern region of Taiwan. This study was approved by the institutional review board (IRB) of Kaohsiung Chang Gung Memorial Hospital with approval number 105-1108C. Informed consent was waived according to IRB regulations. A retrospective study was designed to review all data of patients who were entered into the Trauma Registry System between 1 January 2009 and 31 December 2014. The inclusion criteria were as follows: (1) age ≥20 years, (2) patients suffering from mandibular fracture. Patients with incomplete registered data were excluded from this study. Detailed patient information was retrieved from the Trauma Registry System of our institution and included data regarding age, sex, cause of injury (motorcycle accident, fall accident, motor vehicle accident, struck by/against objects, bicycle accident, and pedestrian injury in a traffic accident), helmet use, the first Glasgow Coma Scale (GCS) during admission in the emergency department, the GCS and associated head injury. Underweight patients were defined as a body mass index (BMI) of <18.5 kg/m^2^ and normal-weight patients with a BMI of <25 but ≥18.5 kg/m^2^, overweight patients with a BMI ≥25 but <30 kg/m^2^, and obese patients with a BMI ≥ 30 kg/m^2^. Multivariate logistic regression were used to compare the category variables and corresponding odds ratios (ORs) and 95% confidence intervals (CIs) were obtained, taking the cause of motorcycle accidents as the reference category. In motorcycle accident, sex, age, helmet-wearing status, and BMI categories were chosen as covariates in multivariate analyses to evaluate the association of helmet-wearing status or BMI categories with the fracture pattern of mandibular fracture. All results are presented as the mean ± standard deviation. A *p*-value less than 0.05 was considered statistically significant.

## 3. Results

From 1 January 2009 to 31 December 2014, the Trauma Registry System included 20,106 hospitalized and registered patients. There were 396 patients that had sustained a mandibular fracture. After excluding 83 patients who aged less than 20 years and one patient who had incomplete data, a total of 503 mandibular fractures suffered by 312 patients were included (Table 1). Of these patients, 207 were men and 105 were women, resulting in a ratio of 2:1. The mean age was 36.5 ± 15.2 years. Motorcycle accident accounted for the major cause of mandibular fracture (76.3%), followed by fall (10.9%), motor vehicle accident (4.8%), and struck by/against objects (4.5%). Bicycle accident and pedestrian injury in a traffic accident only represented 2.2% and 1.3%, respectively, of the cause of injury. These patients with mandibular fractures presented with a GCS of 13.5 ± 3.1 upon arrival at the emergency department, with 11.2% of patients being in a severe comatose condition (GCS ≤ 8). Of the patients with mandibular fractures, 11.5% had cranial fracture, 5.8% had epidural hematoma, 10.3% had subdural hematoma, 10.9% had subarachnoid hemorrhage, 2.6% had intracerebral hematoma, and 4.5% had a cerebral contusion, resulting in 107 of 312 (34.3%) patients suffering a severe head injury, such as cranial fracture or traumatic brain injury. Of the 503 fracture sites of the mandible, condylar neck and head comprised the most common parts of mandibular fractures (32.0%), followed by parasymphysis (21.7%), symphysis (19.5%), angle and ramus (17.5%), and body (9.3%).

**Figure 1:**
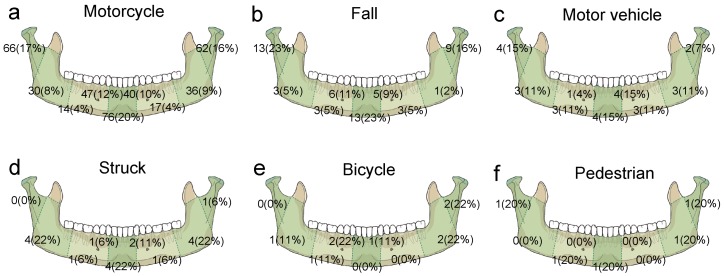
Numbers and percentage of patients per fracture sites of the mandible caused by different trauma. (**a**) motorcycle; (**b**) fall; (**c**) motor vehicle; (**d**) struck; (**e**) bicycle; (**f**) pedestrian.

As shown in the Figure 1 and Table 2, the location of mandibular fractures in motorcycle accident patients presented a profile similar to the one for all patients, as condylar neck and head comprised the most common parts of mandibular fractures (33.0%), followed by parasymphysis (22.4%), symphysis (19.6%), angle and ramus (17.0), and body (8.0%). Compared to motorcycle accidents, motor vehicle accidents resulted in a significant increase in body fractures (OR 3.3, 95% CI 1.24–8.76, *p* = 0.017) and struck injury caused a significant higher number of angle and ramus fractures (OR 3.9, 95% CI 1.48–10.26, *p* = 0.006), but less condylar neck and head fractures (OR 0.1, 95% CI 0.02–0.91, *p* = 0.040). Patients suffering from fractures caused by fall accidents, bicycle accidents, or injuries as a pedestrian had no significant difference in location of mandibular fractures in comparison to fractures caused by motorcycle accidents. In addition, the multivariate analyses (Table 3) revealed that the status of helmet-wearing and body weight were not associated with the location of mandible fractures caused by motorcycle accidents.

## 4. Discussion

In contrast to studies that indicated that motor vehicle accidents and assaults comprised the majority of causes of mandibular fractures [3,4,5,9], our study showed that only 9.3% of mandible fractures were caused by these two actions. Our study revealed that motorcycle accidents accounted for the major cause of mandibular fractures in southern Taiwan. In both the motorcyclist group and the group with all patients, the condylar neck and head were the most frequent location for fractures, followed by the parasymphysis, symphysis, angle and ramus, and body. In addition, motor vehicle accidents caused more body fractures, while struck injuries caused more angle and ramus fractures compared to motorcycle accidents. For motorcycle accidents, the vector of force is often applied on the mandible in anterior–posterior direction, which initiates the force posteriorly to the condyles. The results are in accordance with that reported by Morris et al., who indicated that a high-velocity blunt injury like motorcycle collisions would result in a larger number of condylar fractures, while the mandibular angle is the most common fracture site involved in a low-velocity blunt injury like assault or struck injury [8]. However, in this study, high-velocity blunt injury like motor vehicle accidents showed a 3.3-fold increase in body fractures compared to motorcycle accidents, this observation is not in accordance with the reports from some studies that indicated symphysis and parasymphysis fractures are the most involved anatomic sites [9,18,19]. Because of the structure and anatomic proximity of the mandible as well as its direct connection to the skull base, trauma to the mandible is related to intracranial injury, and traumatic brain injuries were observed in 19% of trauma patients with mandibular fractures [20]. A 75% (12 of 16) rate of concussions associated with isolated mandible fracture has been reported [21]. Similarly, closed head injury has been identified in 32.4% of patients with mandible fractures caused by motor vehicle accidents [22]. It was reported that 16.2% of patients with mandibular fractures fit the indication of neurosurgical treatment [23]. In this study, 34.3% patients with mandibular fractures suffered from concomitant cranial fractures or traumatic brain injury, and therefore physicians should be aware of this when evaluating patients with mandibular fractures.

The protective effect of motorcycle helmets is already well-established in the literature regarding head injury [24,25,26,27] and extensive injury in the face [28]. In a review of 272 helmeted and non-helmeted victims with moderate traumatic brain injury admitted at a referral trauma hospital, the only facial bone fracture with significant association with the type of helmet was the Le Fort fracture [28]. In this study, we did not identify an association of helmet-wearing status and the location of mandible fractures caused by motorcycle accidents. However, the type of helmet (opened helmet or full-face helmet) in the accidents was unknown. In addition, although it has been reported that obese trauma patients experience fewer mandibular fractures than normal bodyweight patients in Taiwan [17,29], we found no significant association between weight of patients and mandibular fracture sites.

Limitations of this study are the retrospective design and the lack of available data regarding conditions including speed, helmet material and design, and prior history of facial bone fracture. Additionally, the number of patients in the study was relatively small, which excluded an in-depth examination of variables, such as the elderly. Furthermore, lack of availability of important confounders regarding the circumstances of the mechanism of injury, exposure data, protection methods, and associated facial bone fractures may result in selection bias. A relatively small population of patients suffered from a bicycle accident or was injured as a pedestrian, making the interpretation or analysis of these groups of patients less meaningful. In addition, patients who had died before arrival at the hospital or at accident scenes were not included in the Trauma Registry Database, thereby creating a selection bias. Finally, this study is limited to one trauma center, thus a selection bias is possible.

## 5. Conclusions

Our study revealed that the anatomic fracture sites of mandible were specifically related to different etiologies. In southern Taiwan, motorcycle accidents accounted for the major cause of mandibular fractures and were associated with the condylar neck and head as the most frequent fracture sites. In contrast, motor vehicle accidents and struck injuries tended to cause more body fracture as well as angle and ramus fracture compared to motorcycle accidents. Furthermore, the status of helmet-wearing and body weight were not associated with the location of mandible fractures caused by motorcycle accidents.

## Figures and Tables

**Table 1:** Demographics and injury characteristics of adult trauma patients with mandible fractures.

Variables	Patients *N* = 312
**Gender (%)**	
Male	207(66.3)
Female	105(33.7)
**Age**	36.5 ± 15.2
**Cause (%)**	
Motorcycle accident	238(76.3)
Fall	34(10.9)
Motor vehicle accident (MVA)	15(4.8)
Struck by/against objects	14(4.5)
Bicycle accident	7(2.2)
Pedestrian	4(1.3)
**GCS**	13.5 ± 3.1
≤8 (%)	35(11.2)
9–12 (%)	23(7.4)
≥13 (%)	254(81.4)
**Associated head injury (%)**	
Cranial fracture	36(11.5)
Epidural hematoma (EDH)	18(5.8)
Subdural hematoma (SDH)	32(10.3)
Subarachnoid hemorrhage (SAH)	34(10.9)
Intracerebral hematoma (ICH)	8(2.6)
Cerebral contusion	14(4.5)
Cervical vertebral fracture	4(1.3)
**Site of mandibular fracture (%)**	*n* = 503
Symphysis	98(19.5)
Parasymphysis	109(21.7)
Body	47(9.3)
Angle and Ramus	88(17.5)
Condylar neck and Head	161(32.0)

*N* = number of patients, *n* = number of fractures.

**Table 2:** The association between cause of trauma and sites of mandibular fractures.

**Variables**	**Fall *n* = 56 (II)**		**MVA *n* = 27 (III)**		**Struck by/against *n* = 18 (IV)**		**Bicycle *n* = 9 (V)**		**Pedestrian *n* = 5 (VI)**	
Symphysis	13(23.2)		4(14.8)		4(22.2)		0(0.0)		1(20.0)	
Parasymphysis	11(19.6)		5(18.5)		3(16.7)		3(33.3)		0(0.0)	
Body	6(10.7)		6(22.2)		2(11.1)		1(11.1)		1(20.0)	
Angle and Ramus	4(7.1)		6(22.2)		8(44.4)		3(33.3)		1(20.0)	
Condylar neck and Head	22(39.3)		6(22.2)		1(5.6)		2(22.2)		2(40.0)	
**Variables**	***OR (95% CI)***	***p***	***OR (95% CI)***	***p***	***OR (95% CI)***	***p***	***OR (95% CI)***	***p***	***OR (95% CI)***	***p***
	**II vs. I**		**III vs. I**		**IV vs. I**		**V vs. I**		**VI vs. I**	
Symphysis	1.2(0.64–2.42)	0.527	0.7(0.24–2.13)	0.545	1.2(0.38–3.66)	0.784	-	0.999	1.0(0.11–9.34)	0.982
Parasymphysis	0.8(0.421.71)	0.639	0.8(0.29–2.14)	0.637	0.7(0.20–2.45)	0.567	1.7(0.42–7.06)	0.445	-	0.999
Body	1.4(0.55–3.48)	0.492	3.3(1.24–8.76)	0.017	1.4(0.32–6.55)	0.637	1.4(0.17–11.89)	0.735	2.9(0.31–26.56)	0.351
Angle and Ramus	0.4(0.13–1.07)	0.068	1.4(0.54–3.59)	0.491	3.9(1.48–10.26)	0.006	2.4(0.60–10.00)	0.215	1.2(0.13–11.09)	0.860

*n* = number of fractures.

**Table 3:** The association between helmet use and body mass index (BMI) in motorcycle accidents and sites of mandibular fractures.

**Variables**	**Symphysis *n* = 76**		**Parasymphysis *n* = 87**		**Body *n* = 31**		**Angle and Ramus *n* = 66**		**Condyle** ** and Head *n* = 128**	
Age	32.2 ± 10.9		32.2 ± 12.2		39.0 ± 16.4		35.5 ± 17.1		33.6 ± 13.4	
Gender (M/F)	29/47		30/57		7/24		24/42		60/68	
BMI classification										
Obese	4(5.3)		8(9.2)		0(0.0)		3(4.5)		5(3.9)	
Overweight	16(21.1)		19(21.8)		9(29.0)		14(21.2)		22(17.2)	
Underweight	7(9.2)		5(5.7)		2(6.5)		3(4.5)		11(8.6)	
Normal	45(59.2)		53(60.9)		20(64.5)		43(65.2)		83(64.8)	
Unknown	4(5.3)		2(2.3)		0(0.0)		3(4.5)		7(5.5)	
Helmet use										
Yes	58(76.3)		71(81.6)		29(93.5)		50(75.8)		112(87.5)	
No	13(17.1)		14(16.1)		2(6.5)		13(19.7)		14(10.9)	
Unknown	5(6.6)		2(2.3)		0(0.0)		3(4.5)		2(1.6)	
**Variables**	**Symphysis**		**Parasymphysis**		**Body**		**Angle and Ramus**		**Condyle and Head**	
	***OR (95% CI)***	***p***	***OR (95% CI)***	***p***	***OR (95% CI)***		***OR (95% CI)***	***p***	***OR (95% CI)***	***p***
BMI classification						BMI classification				
Obese	1.2(0.38–3.91)	0.739	1.9(0.72–5.21)	0.192	-	Obese	1.2(0.38–3.91)	0.739	1.9(0.72–5.21)	0.192
Overweight	1.3(0.65–2.51)	0.486	1.2(0.66–2.31)	0.502	1.0(0.41–2.35)	Overweight	1.3(0.65–2.51)	0.486	1.2(0.66–2.31)	0.502
Underweight	1.5(0.56–4.05)	0.418	1.0(0.34–2.70)	0.933	1.3(0.28–6.38)	Underweight	1.5(0.56–4.05)	0.418	1.0(0.34–2.70)	0.933
Helmet use						Helmet use				
Yes	0.6(0.29–1.22)	0.158	0.9(0.45–1.84)	0.803	3.9(0.87–17.26)	Yes	0.6(0.29–1.22)	0.158	0.9(0.45–1.84)	0.803

*n* = number of fractures.
